# Genomic analysis–integrated whole-exome sequencing of neuroblastomas identifies genetic mutations in axon guidance pathway

**DOI:** 10.18632/oncotarget.18079

**Published:** 2017-05-23

**Authors:** Yuanyuan Li, Miki Ohira, Yong Zhou, Teng Xiong, Wen Luo, Chao Yang, Xiangchun Li, Zhibo Gao, Rui Zhou, Yohko Nakamura, Takehiko Kamijo, Yasuhiko Kaneko, Takeshi Taketani, Junichi Ueyama, Tatsuro Tajiri, Hongyan Zhang, Jian Wang, Huanming Yang, Ye Yin, Akira Nakagawara

**Affiliations:** ^1^ Life Science Research Institute, Saga Medical Center Koseikan, Saga, Japan; ^2^ Division of Biochemistry and Innovative Cancer Therapy, Chiba Cancer Center Research Institute, Chiba, Japan; ^3^ Division of Cancer Genomics, Chiba Cancer Center Research Institute, Chiba, Japan; ^4^ Research Institute for Clinical Oncology, Saitama Cancer Center, Saitama, Japan; ^5^ BGI-Shenzhen, Shenzhen, China; ^6^ Department of Pediatrics, Shimane University School of Medicine, Shimane, Japan; ^7^ Division of Pediatrics and Perinatology, Tottori University School of Medicine, Tottori, Japan; ^8^ Department of Pediatric Surgery, Kyoto Prefectural University of Medicine, Kyoto, Japan; ^9^ James D. Watson Institute of Genome Science, Hangzhou, China

**Keywords:** whole-exome sequencing, array CGH, neuroblastoma, familial neuroblastoma, genomic diversity

## Abstract

Neuroblastoma (NB) is a childhood solid malignant tumor originating from precursor cells of the peripheral nervous system. We have previously established a risk classification system based on DNA copy number profiles. To further explore the pathogenesis of NBs in distinct risk groups, we performed whole-exome sequencing analysis of 57 primary and 7 recurrent/metastatic tumors with unique chromosomal aberration profiles as categorized by our genomic sub-grouping system. Overall, a low frequency of somatic mutations was found. Besides *ALK* (4/64, 6.3%), *SEMA6C*, *SLIT1* and *NRAS*, genes involved in the axon guidance pathway, were identified as recurrently mutated in 6 of 64 tumors (9.4%). Pathway enrichment analysis revealed enrichment of 25 mutated genes in the mitogen-activated protein kinase (MAPK) pathway, 13 genes in the Wnt pathway, and 12 genes in the axon guidance pathway. Genomic analyses demonstrated that primary and matched recurrent or metastatic tumors obtained from sporadic and monozygotic twin NBs were clonally related with variable extents of genetic heterogeneity. Monozygotic twin NBs displayed different evolutionary trajectories. These results indicate the involvement of the axon guidance, MAPK and Wnt pathways in NB and demonstrate genomic diversity with NB progression.

## INTRODUCTION

Neuroblastoma (NB) is one of the most common solid tumors in children, which originates from precursor cells of the peripheral (sympathetic) nervous system. One striking characteristic of NB is the heterogeneous biological and clinical behaviors exhibited by individual tumors. Patients over 1 year of age usually suffer from disseminated disease at diagnosis and have a poor outcome despite intensive multimodal treatment [1–3]. Currently, NB patients are stratified into low-, intermediate-, or high-risk categories based on a risk assessment of well-defined prognostic factors including the age at diagnosis, International Neuroblastoma Staging System (INSS) stage, tumor histopathology, *MYCN* amplification status and tumor DNA ploidy. The 5-year survival rates (SRs) for NB are approximately 95% for the low-risk group and 80–90% for the intermediate-risk group, but only 30–50% for high-risk NB.

The extreme clinical heterogeneity of NB reflects the complexity of genetic and genomic events associated with disease development and progression. To date, several genomic changes that correlate with an unfavorable prognosis for NB have been identified. *MYCN* oncogene amplification is present in approximately 20% of NB patients and correlates with tumor progression, advanced disease stages and poor outcome [1, 2]. Additionally, chromosomal aberrations such as 1p loss, 11q loss and 17q gain have been shown to predict poor patient outcome [1, 2]. Several genetic mutations associated with NB have also been identified. Germ-line mutations in paired-like homeobox 2B (*PHOX2B*) have been discovered in a minority of familial NB patients and account for their predisposition to NB [4–8]. Activating mutations in the tyrosine kinase domain of the anaplastic lymphoma kinase (*ALK*) oncogene are acquired hereditarily or somatically in familial and sporadic NB cases, respectively, and account for disease susceptibility as well as disease progression [9–12]. Recent studies using next-generation sequencing (NGS) approaches have identified mutations in the *ATRX* gene that are strongly associated with late onset age of NB [13, 14], a high frequency of chromothripsis in stage 3 and 4 tumors (18%) and frequent mutations in the Rac/Rho pathway genes that guide neuritogenesis [15, 16], and recurrent *TERT* rearrangements in high-risk NBs (24%) [17, 18]. Chromosomal deletions and sequence alterations in the *ARID1A/1B* genes, which regulate chromatin remodeling, have also been detected in 11% of NB patients [19].

We have previously established a genomic subgrouping system based on array CGH analysis for the risk stratification of neuroblastoma [20, 21]. To further identify novel somatic mutations linked to tumorigenesis and different outcomes in NB, in this study we focused on the two major subgroups, Ss and P1a, with favorable and unfavorable clinical outcomes, respectively, to perform whole-exome sequencing (WES) analysis using the Illumina platform. Our results indicate that the axon guidance, MAPK and Wnt pathways may be involved in molecular behavior of NB. Furthermore, we describe the genetic heterogeneity within NB tumors and evolutionary trajectories of monozygotic twin NBs.

## RESULTS

### Genomic subgrouping and risk stratification of NBs

**Figure 1:**
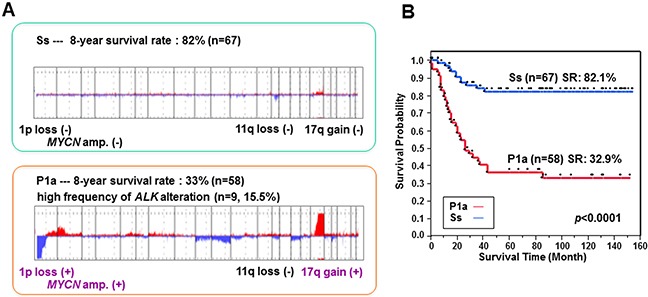
Genomic profiles of Ss and P1a subgroups and their association with overall survival in NB **(A)** Genomic aberration profiles of Ss and P1a subgroups identified by array CGH analysis. The 8-year survival rate was calculated using a cohort of 343 NBs. **(B)** Kaplan–Meier survival curves drawn for Ss versus P1a subgroups. Survival distributions were compared using the log-rank test.

We have previously established a genomic sub-grouping system based on array CGH analysis for the risk stratification of neuroblastoma in 236 primary NBs [18, 19]. Using this system, we have further investigated an additional independent cohort of 107 primary NBs for their risk classification. Array CGH analysis revealed three major chromosomal aberration profiles in the 343 total NBs examined: a silent (S) pattern almost without any chromosomal aberrations; a partial gains and/or losses (P) pattern; and a whole gains and/or losses (W) pattern. Each group was further classified into several subgroups on the basis of clinical outcome and known genomic signatures, including *MYCN* amplification, 1p loss, 11q loss and 17q gain. Consequently, the S group was divided into Ss (s, *MYCN* non-amplification) and Sa (a, *MYCN* amplification) subgroups according to the *MYCN* copy number, and the P group into P1 (1p loss and 17q gain), P2 (1p loss, 11q loss and 17q gain), P3 (11q loss and 17q gain) and P4 (17q gain) subgroups, each of which was further split into s and a subsets in light of *MYCN* amplification status. The W group was characterized by whole chromosomal gains and/or losses, especially the gain of chromosome 17, and rarely exhibited *MYCN* amplification. Among them, the major subgroups with high frequency are Ss (n = 67, 20%), P1a (n = 58, 17%) and W4s (n = 87, 25%). Notably, the Ss subgroup had an overall 8-year SR of 82%, whereas the P1a subgroup, which possessed genomic signatures predicting unfavorable outcome in NB including 1p loss, 17q gain and *MYCN* amplification, had an overall 8-year SR of 33% (Figure 1). Moreover, P1a tumors harbored a high frequency of aberrations in the *ALK* gene (9/58, 15.5%; including mutation (n = 6) and amplification (n = 3)).

### Whole exome sequencing analysis

To identify novel somatic mutations linked with tumorigenesis and different outcome in NB, in this study we employed a WES approach to 56 paired NBs (57 primary tumors and 7 recurrent/metastatic tumors; median age, 19 months; Supplementary Table 1) with a focus on Ss (n = 21) and P1a (n = 17) subgroups. All samples were selected on the basis of the availability of high-quality and sufficient DNA for WES. Our sample set contained 51 sporadic and 5 familial background NBs. Additionally, 7 recurrent/metastatic tumors from individual cases were examined so as to investigate genome heterogeneity between primary and recurrent/metastatic tumors.

**Figure 2:**
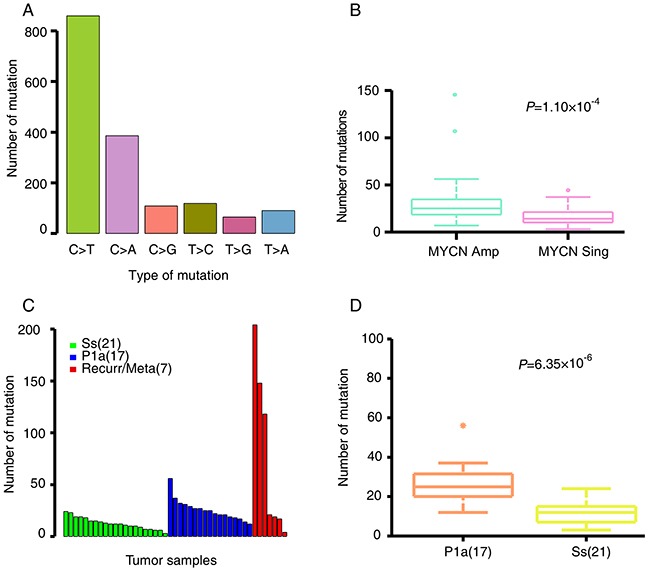
NB somatic mutation characteristics **(A)** The mutation spectra of somatic single nucleotide mutations. **(B)** Average number of somatic alterations in NB patients with *MYCN* amplification (*MYCN* Amp) and *MYCN* non-amplification (*MYCN Sing*). **(C)** The number of somatic mutations in Ss, P1a and recurrent/metastatic tumors. **(D)** Average number of somatic alterations in Ss and P1a subgroups. The number of patients per group is shown in parentheses. Box plot in R software was used to create the graph. Permutation test in R software was used to assess statistical difference.

In total, 64 tumors and their matched blood-derived DNA samples were examined by WES using an Illumina Hiseq2000 platform with a minimum sequencing depth of 75X per case. We identified 1685 candidate somatic mutations in 1263 genes from 64 tumors, including 417 silent mutations, 1132 missense, 60 nonsense, 19 splice-site changes and 57 insertions or deletions (indels). On average, each tumor harbored 26.3 coding mutations (19.8 non-silent mutations comprising 18.9 substitutions and 0.9 indels) with a wide range of 3–204 (Table 1 and Supplementary Table 2). Mutation spectrum analysis showed that single-nucleotide mutations were predominantly C–T substitutions (Figure 2A), similar to previous observations for NB [22] and adult tumors [23]. Moreover, the mutation rate is strongly correlated with *MYCN* amplification (*P* = 1.10×10^−4^; Figure 2B).

**Table 1:** Summary of somatic mutations identified in 64 NB tumors

Mutation type	Number	Description
Silent	417	Mutation causing no change in the amino acid
Missense	1132	Mutation encoding a different amino acid
Nonsense	60	Mutation encoding a premature stop codon
Splice-site change	19	Mutation within 2bp from splicing site
Indel	57	Small insertion or deletion

The recurrent/metastatic tumors harbored the most somatic mutations, as compared with Ss and P1a primary tumors (Figure 2C). P1a tumors possessed significantly more somatic mutations than Ss tumors (permutation test, P = 6.35×10^−6^; Figure 2C and 2D). Of the 1268 non-silent somatic mutations, only 211 were discovered in 21 primary and 2 recurrent Ss tumors (mean, 9 mutations per tumor; range, 2–18). In contrast, 520 mutations were detected in 17 primary and 2 recurrent/metastatic P1a tumors (mean, 27 mutations per tumor; range, 2–160), whereas recurrent and metastatic tumors harbored the highest number of somatic mutations (mean, 57 mutations per tumor; range, 0–174). Notably, four recurrent/metastatic tumors show fewer mutations that are comparable to that of the Ss primary tumors. Of these, two tumors (NB56-T2 and NB57-T2) were obtained from the metastatic lesions along with the primary tumors from the twin patients with stage 4s NB. Their genomic profiles are both W4s characterized by whole chr17 gain without 1p loss, 11q loss and *MYCN* amplification. Considering that the overall 5-year SR of the W4s subgroup in our dataset is 97% [20, 21] and that the two patients are alive, such a lower number of somatic mutations even in the metastatic lesions account for their favorable outcomes. The other two tumors (NB37-T2 and NB51-T2) are recurrent ones that were extracted after neoadjuvant therapy. In view that there are significant differences in genomic profiles between the primary and recurrent tumors (P1a in NB37-T1 and Ss in NB37-T2, Sa in NB51-T1 and Ss in NB51-T2), fewer mutations in the two recurrent tumors may be attributed to the loss of viable tumor cell content due to neoadjuvant therapy.

### Potential genes and pathways

We detected 27 genes with non-silent somatic mutations occurring in at least two NB cases (Table 2). The mRNA expression levels of these genes and their association with overall survival in NB were verified using our expression data and two independent NB microarray analysis datasets (Kocak - 649 - custom - ag44kcwolf and SEQC - 498 - RPM - seqcnb1) in the R2: microarray analysis and visualization platform (http://r2.amc.nl), except *OR52R1* whose data were absent in one of the datasets. Of these genes, *ALK* mutations, which were all restricted to the kinase domain, were found in 7.0% (4/57) of the primary tumors, consistent with the frequencies identified in several large NB cohorts using conventional sequencing method [9–12]. Apart from *ALK*, mutations in *TTN* and *EMR1* were detected in 5 and 3 out of 64 NB tumors (7.8% and 4.7%), respectively. *PCDHGA4*, *PLCH2*, *NRAS* and *SEMA6C* harbored mutations at the same sites in two independent cases. Gain-of-function mutations to *NRAS* have been described in many adult tumors as well as stage 4 NBs recently [14]. In our dataset, the p.Gln61Lys mutation was identified in two P1a tumors: one primary tumor from patient NB43 and the other a recurrent tumor from NB53 (-T2). Both individuals died of disease. Notably, this mutation was also identified in 3 of 25 NB cell lines. Somatic mutations in *PLCH2* (p.Thr177Met), *PCDHGA4* (p.Asp536Tyr) and *SEMA6C* (p.Leu348Arg) commonly appeared in NB56-T1/T2 and NB57 (patients NB56 and NB57 are twins) at stage 4s with the W4s genomic profile. None of the 27 genes were commonly mutated in two Ss tumors. However, *SLIT1*, *SYNE1* and *VPS13C* were mutated in two primary P1a tumors, respectively.

**Table 2:** Summary of non-silent mutations occurring in ≥ 2 cases across 64 NB tumors

Gene symbol	Mutation site	Number of cases	mRNA expression*
TTN	7	5	H>L
MUC16	5	5	H<L
ALK	4	4	H<L
EMR1	3	3	H=L
SAMD9L	3	2	H>L
RAD54L2	2	2	H<L
RBMX	2	2	H<L
TENC1	2	2	H>L
VPS13C	2	2	H>L
UTRN	2	2	H>L
TRIM42	2	2	H=L
SYNE1	2	2	H>L
SLIT1	2	2	H<L
SDSL	2	2	H<L
OR52R1	2	2	N
FMO3	2	2	H>L
ENTHD1	2	2	H=L
CYP4F11	2	2	H=L
CMYA5	2	2	H=L
LILRA3	2	2	H=L
NPAP1	2	2	H=L
POLN	2	2	H=L
PCDHGA4	1	2	H>L
PLCH2	1	2	H>L
SEMA6C	1	2	H>L
ZNF304	1	2	H=L
NRAS	1	2	H=L

*indicates the correlation of mRNA expression level with overall survival as determined by Kaplan-Meier analysis using two independent microarray analysis datasets from the open-access R2 interface (Kocak - 649 - custom - ag44kcwolf and SEQC - 498 - RPM - seqcnb1). For PCDHGA4, the correlation was evaluated using Versteeg - 88 - MAS5.0 - u133p2 dataset instead of Kocak - 649 - custom - ag44kcwolf dataset, since this gene does not exist in the Kocak - 649 dataset. H, high expression; L, low expression; H=L, there is no significant difference in overall survival between low and high expression subsets; H>L, high expression is significantly associated with good overall survival; H<L, low expression is significantly associated with good survival; N, no data in the datasets used.

**Figure 3:**
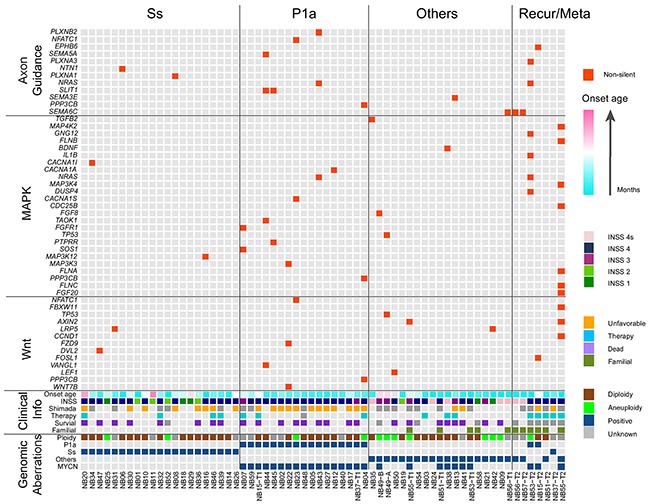
Landscape of genomic and genetic alternations in 64 NB samples All samples used in our WES approach were divided into Ss, P1a, others and recurrent/metastatic (Recur/Meta) subgroups. Genes with non-silent mutation that were involved in the axon guidance, MAPK and Wnt pathways are shown in red blocks. Clinical information included age at diagnosis (increasing age, blue to pink spectrum), INSS stage, Shimada classification (unfavorable, orange; unknown, grey), presence or absence of preoperational therapy (yes, blue; unknown, grey), survival status (dead, violet; unknown, grey) as well as familial background (yes, yellow green). Genomic aberrations included DNA ploidy (aneuploidy, green; diploidy, brown), genomic subgroups of Ss, P1a and others (positive, blue) and *MYCN* amplification (positive, blue).

Intriguingly, *SEMA6C*, *SLIT1* and *NRAS* amid 27 recurrently mutated genes were involved in the axon guidance pathway (6 out of 64 NB tumors, 9.4%). Further pathway enrichment analysis of our 1268 non-silent mutation gene set identified altogether 12 genes belonging to the axon guidance pathway (12 out of 64 NB tumors, 18.8%; Figure 3, Table 3 and Supplementary Table 3).

**Table 3:** List of mutated genes involved in the axon guidance pathway

Gene	Chr.*	Nucleotide (cDNA)	Amino acid	No. tumors	Base samples
PLXNB2	22	c.G989A	p.C330Y	1	NB43
NFATC1	18	c.A1349T	p.Q450L	1	NB23
EPHB6	7	c.G130T	p.A44S	1	NB15-T2
SEMA5A	5	c.G3097A	p.A1033T	1	NB44
PLXNA3	X	c.G4988A	p.G1663D	1	NB53-T2
NTN1	17	c.C1105A	p.L369I	1	NB06
PLXNA1	3	c.G3806A	p.R1269H	1	NB08
NRAS	1	c.C181A	p.Q61K	2	NB43, NB53-T2
SLIT1	10	c.C2128A, c.G863T	p.Q710K, p.C288F	2	NB44, NB45
SEMA3E	7	c.C1903T	p.P635S	1	NB13
PPP3CB	10	c.C1375T	p.R459X	1	NB04
SEMA6C	1	c.T1043G	p.L348R	2	NB56, NB57

*Chr., chromosome.

**Figure 4:**
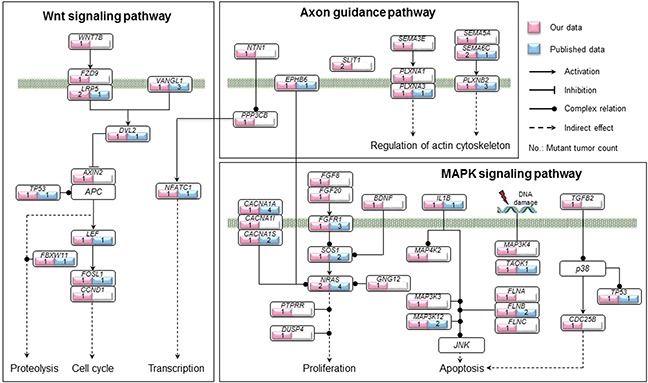
Summary of genetic alterations involved in the axon guidance, MAPK and Wnt pathways identified in our study Genes with non-silent mutations that were involved in the axon guidance, MAPK and Wnt pathways are shown based on KEGG pathway database. The genes identified in our study are indicated in pink and those identified in previous studies in 422 primary NBs in total [14, 15, 17, 19, 22, 45] are indicated in blue, along with the count of mutant tumors in each gene. The solid circle indicates complex regulation relation.

Using Polyphen-2 [24] and SIFT, we assessed the functional impact of missense mutations found in our dataset, and defined those with possible and probable damaging scores along with nonsense mutations, splice site mutations and indels as high functional impact (HiFI) mutations [25]. These data were subjected to pathway enrichment analysis by means of the KEGG pathway database to identify the genes enriched in cancer-related signaling pathways. Pathways in which mutations were present in more than 10% of our samples are shown in Supplementary Table 3. Among the 1268 non-silent mutations, 13 mutations in 12 genes were enriched in axon guidance pathway, and 14 mutations in 13 genes were enriched in the Wnt signaling pathway (Figure 3 and Supplementary Table 3). The vast majority of these aberrations were HiFI mutations. Moreover, 8 of the 12 mutated genes involved in the axon guidance pathway and 4 of the 13 genes involved in the Wnt pathway were detected in P1a tumors. Additionally, 25 genes with 25 mutations were involved in the MAPK pathway (Figure 3 and Supplementary Table 3), and 12 of these were present in P1a tumors, whereas only 2 were found in Ss tumors (47.4% (9/19) in P1a vs. 8.7% (2/23) in Ss, *P* = 0.01297; Table 4). Mutated genes identified in our study that were involved in the axon guidance, MAPK, and Wnt pathways are summarized in Figure 4. Notably, PPP3CB, a catalytic subunit of calcineurin, was involved with the MAPK, Wnt and axon guidance pathways in our dataset. Intriguingly, the mutation found in *PPP3CB* at p.Arg459 is a nonsense mutation located ahead of the C-terminal autoinhibitory domain and thus results in a constitutively active form of the PPP3CB protein.

**Table 4:** Distribution of 25 genes involved with the MAPK pathway in different genomic subgroups

Genomic group	No. genes	No. tumors	Total no. tumors	Appearance (%)	Classification
**P1a**	12	9	19	47.37	8 primary 1 metastatic
**Ss**	2	2	23	8.7	2 primary
**Others**	11	5	22	22.73	4 primary 1 recurrent

### Intra-tumor heterogeneity in sporadic NB tumors and evolution of monozygotic twin NBs

Several previous studies have documented intra-tumor heterogeneity across multiple spatially- or temporally-separated cancer-tissue samples [26–28]. We included 4 recurrent and 3 metastatic tumors obtained from 2 stage 4s and 5 stage 4 cases, respectively, in our samples. Different genomic profiles between primary and recurrent/metastatic tumors were observed in some of these cases. Therefore, we next explored tumor evolution and mutational dynamics in NB genomes by applying ABSOLUTE1.2 analysis to infer cancer-cell fractions (CCFs) of somatic point mutations and copy number aberrations. We used this CCF analysis to build phylogenetic trees for the tumors involving relapse/metastasis.

**Figure 5:**
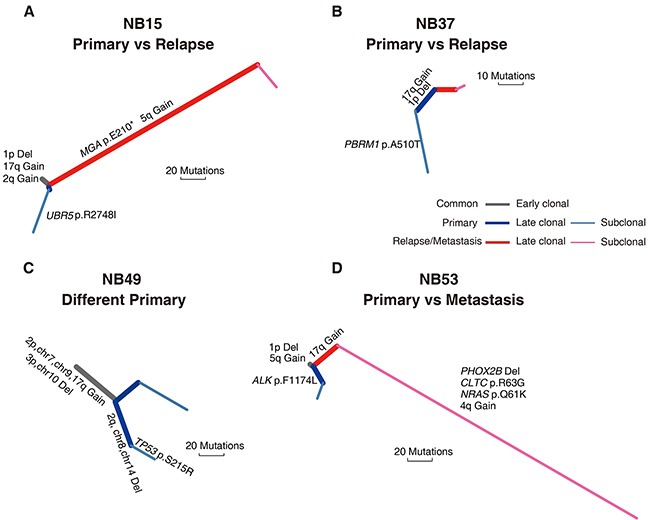
Phylogenetic trees of four spatially or temporally separated NB tumor pairs Two primary vs. relapsed tumor pairs from NB15 **(A)** and NB37 **(B)**, respectively, two primary tumor specimens with different macroscopic appearance excised from the same tumor mass in NB49 **(C)** and a primary vs metastasis tumor pair from NB53 **(D)** were analyzed for tumor evolution and mutational dynamics. Branches with different colors indicate different stages of evolution (gray, shared by all samples; blue, primary tumor; red, recurrent/metastatic tumor). Potentially oncogenic alterations are annotated on each branch. Branch lengths are normalized by somatic point mutations.

NB15 and NB37 had paired relapse tumors after undergoing neoadjuvant therapy. The NB15 recurrent tumor possessed markedly more non-silent mutations than the primary lesion (73 vs. 19). NB15 had copy number alterations such as 1p deletion and 2q, 17q amplification that were shared by both the primary and relapse tumors. However, we identified a nonsense mutation in *MGA* (p.Glu210*), a tumor suppressor gene that interacts with *MAX* [29], only in the NB15 relapse tumor. This relapse tumor also harbored a broad amplification in *TRIO* that was not present in the primary lesion (Figure 5A). We did not detect any common mutations between the NB37 primary and relapse tumors (Figure 5B). This may be because of the low quantity of the relapse sample, as it exhibited a lower-grade copy number change and a lower mutation frequency than the primary sample (Supplementary Figure 1 and Supplementary Table 2).

NB49 did not receive any neoadjuvant treatment and had two specimens with different macroscopic appearance excised from the same tumor mass (NB49-A and NB49-B). Our array CGH analysis showed that NB49-A and NB49-B had P4s (unamplified *MYCN*, NB49-A) and P4a (*MYCN*: ∼20 copies, NB49-B) genomic profiles, respectively. These two primary samples shared large-scale copy number events, suggesting an early macroevolution event in the evolutionary trajectory of this case. However, a missense mutation of *TP53* (p.Ser215Arg) was only observed in NB49-A (Figure 5C), indicating a parallel evolution in clonal expansion of tumor cells.

NB53 did not receive neoadjuvant therapy and the primary tumor (NB53-T1) presented an Sa+1pL genomic profile (*MYCN* amplification and 1p loss but no 17q gain nor 11q loss), while the liver metastatic tumor obtained 14 months after therapy (NB53-T2) exhibited a P1a pattern. We observed a significantly higher mutation incidence (148 vs. 17) and somatic copy number aberration burden in the metastatic tumor compared with the primary lesion. These variations could be attributed to subsequent evolution of the metastatic tumor, as we identified many subclonal mutations that were not shared with the primary tumor. The metastatic tumor harbored a *PHOX2B* deletion and a missense mutation of *NRAS* (p.Gln61Lys), whereas an *ALK* mutation (p.Phe1174Leu) was detected only in the primary tumor (Figure 5D). These four cases illustrate the heterogeneity of NB tumors, and suggest that the tumor cells with partial genomic aberrance and *MYCN* amplification might have greater potential for metastasis.

**Figure 6:**
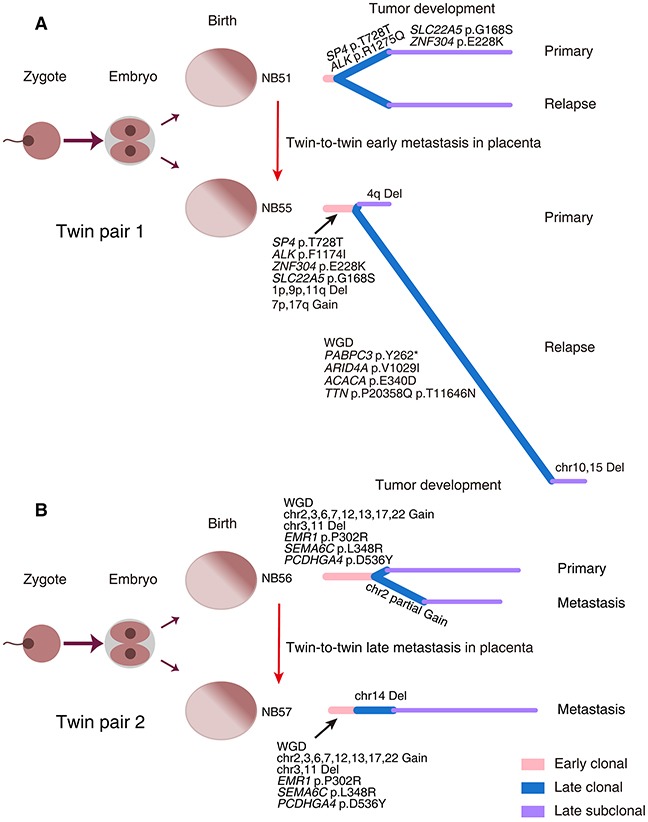
Evolutionary trajectories of monozygotic twin NBs Two pairs of monozygotic twins with NB (Twin pair 1: NB51 and NB55 **(A)**; Twin pair 2: NB56 and NB57 **(B)**) were analyzed for tumor evolution. The left panels depict the zygote development in monozygotic twins. The right panels show the phylogenetic trees inferred for each case. WGD, whole-genome doubling; Del, deletion.

Samples from monozygotic twins with NB are rare, and two pairs were included in our samples. Twin pair 1 (NB51 and NB55) had a 3-year gap between the age at diagnosis. These two cases both relapsed 5 months after therapy. NB51 had an identical mutation complement between the primary and relapsed tumors, whereas NB55 exhibited a higher mutation and somatic copy number alteration (SCNA) burden in the relapse tumor. This complex phenotype may be a result of whole-genome doubling (WGD) in the NB55 relapse tumor leading to increased chromosomal instability. Twin pair 2 (NB56 and NB57) were both diagnosed at 4 months of age; NB56 had primary and metastatic lesions, whereas NB57 only manifested a metastatic liver lesion without a primary site. All specimens from the twins (NB56-T1/T2, NB57-T2) exhibited almost identical copy number alterations. WGD was also observed in all samples of this twin pair. Previous studies have proposed an *in utero* twin-to-twin metastasis model for cancer development in monozygotic twins [30, 31]. Our results provide further evidence supporting this model, as twin pair 1 shared mutations in *SP4* (p.Thr728Thr) and *ZNF304* (p.Glu228lys), and twin pair 2 shared mutations in *EMR1* (p.Pro302Arg), *SEMA6C* (p.Leu348Arg) and *PCDHGA4* (p.Asp536Tyr). Considering the extensive genetic differences between NB51 and NB55, an early metastasis may have occurred in this twin pair during fetal development, with further distinct molecular alterations acquired after birth (Figure 6A). In contrast, twin pair 2 might have experienced a late metastasis since the degree of genetic difference between the lesions was very small (Figure 6B).

## DISCUSSION

Here, we report the genetic mutation landscape of 56 paired Japanese NBs investigated using a WES approach. Unlike adult solid tumors, we found a remarkably smaller mutation spectrum with a very low frequency of recurrent somatic mutations in our sample set (Tables 1 and 2). This is consistent with several previous findings in NB [13–15, 19]. Similarly, medulloblastoma, another major malignant solid tumor of childhood that originates from the cerebellum, has been described to harbor fewer somatic mutations compared with the adult solid tumors [32]. In concert with these studies, our current findings demonstrate a genetic difference between childhood and adult solid tumors. In comparison with genetic alterations, structural chromosomal aberrations are more frequent in NB, especially aggressive tumors.

Our risk stratification system based on array CGH analysis has categorized NBs into three risk groups that have distinct genomic profiles and different prognoses. In this study, we focused on the Ss and P1a major subgroups, which had overall 8-year SRs of 82% and 33%, respectively, to identify specific genetic alterations associated with NB tumorigenesis and different outcome. Surprisingly, the Ss subgroup almost without any genomic abnormalities possessed very few somatic mutations, contrasting sharply with the P1a subgroup. These findings suggest that genomic and genetic aberrations detected by the current analyses might not be the leading cause of tumorigenesis in Ss tumors, and that the extremely low frequency of somatic mutations in the Ss subgroup might contribute to the favorable outcome of Ss tumors. Investigating the role of other mechanisms such as epigenetic alteration will be expected to provide new insights into tumorigenesis in the Ss subgroup. Conversely, the P1a subgroup is characterized by partial chromosomal gains/losses including 1p loss and 17q gain as well as *MYCN* amplification. The existence of partial chromosomal structure alterations demonstrates genomic instability, which may lead to genetic instability. *MYCN* amplification is regarded as a consequence of genomic instability in NB [33]. In turn, overexpression of N-Myc protein resulted from *MYCN* amplification may elicit further genetic instability. Actually, c-Myc, another member of the Myc family, has been demonstrated to promote genetic instability by suppressing DNA double-strand break repair [34–36]. Therefore these features might together account for the relatively higher mutation frequency in P1a tumors. Supporting this notion, the frequency of somatic mutations was remarkably higher in tumors with *MYCN* amplification in our dataset (Figure 2B). This relatively higher frequency of somatic mutations in P1a tumors might contribute to their aggressive behavior and poor outcome.

Intriguingly, we observed differences in the genomic profiles of two specimens isolated from different regions of a single primary tumor mass (P4s in NB49-A and P4a in NB49-B) that did not receive any neoadjuvant therapy. Similar differences were present in a primary/metastatic tumor pair (W4a in NB53-T1 and P1a in NB53-T2). Our phylogenetic analyses further document the existence of intra-tumor heterogeneity and clonal evolution in these NBs (Figure 5). Most recently, similar chromosomal copy number discrepancies between primary and relapse NBs have been reported. Two relapse NB lesions harbored *de novo* chromosome 9p loss and *MYCN* amplification, respectively, compared with their paired primary tumors [28]. These phenomena imply that NB tumor cells with partial genomic aberrance and *MYCN* amplification might have increased metastatic potential. In support, our recurrent/metastatic tumors had the highest number of somatic coding mutations (Figure 2C). Similar results have been recently observed in a cohort of 16 paired primary and relapse NBs [28]. Additionally, we provide the first example of genetic evidence for the twin-to-twin *in utero* metastasis model for cancer development between monozygotic twin NBs (Figure 6).

Remarkably, *SEMA6C*, *SLIT1* and *NRAS* amid the 27 recurrent genes were involved in the axon guidance pathway (6/64, 9.4%). In total, we identified 12 genes with non-silent mutations belonging to this pathway in our dataset (Figures 3 and 4, Table 3), 7 of which were present in P1a tumors (Supplementary Table 2). Notably, 12 of 16 somatic mutations found in these genes were predicted to be HiFI mutations (Supplementary Table 3), strongly implying that these mutations have a functional impact on axon guidance signaling. The axon guidance pathway modulates normal neuronal migration and positioning in the developing nervous system. Accumulating evidence demonstrates that the pathway plays a pivotal role in regulating cancer cell growth, survival, invasion and angiogenesis in a wide variety of cancers [37–39]. Not only have abnormal expression levels of some constituent molecules been widely observed in various cancers, but functional mutations in these genes have been also increasingly identified in multiple human cancers [40–42]. These findings indicate that genetic aberrations in this pathway may contribute to both cancer pathogenesis and cancer cell biological behavior. The extracellular domain of SEMA6C can inhibit axonal extension of nerve growth factor-differentiated PC12 cells and induce the growth cone collapse of chicken dorsal root ganglia, rat hippocampal neurons, and rat cortical neurons in a dose-responsive manner [43]. Intriguingly, the mutation in *SEMA6C* identified in our NB samples was present in the extracellular SEMA domain, suggesting that the mutation might affect the function of SEMA6C. Additionally, two somatic mutations were identified in the N-terminal region of SLIT1, a fragment responsible for both axon guidance and neuronal migration signaling [44]. Multivariate correlation analysis revealed a strong linear correlation between *MYCN* and *SLIT1* expression levels in our NB sample set (data not shown). Elucidating the functional effects of these *SLIT1* mutations will provide important insights into the role of SLIT1/ROBO signaling in NB.

The p.Gln61Lys gain-of-function mutation in *NRAS* was detected in two P1a tumors and 3 of 25 NB cell lines in our dataset. Consistently, *NRAS* has been recently identified as one of several genes mutated at a significant frequency in a cohort of stage 4 NBs [14]. NRAS functions in both the axon guidance and MAPK pathways. 25 genes were enriched in the MAPK pathway in our dataset, 21 of which were concentrated in primary and recurrent/metastatic tumors with *MYCN* amplification (12 of 16 cases; Figure 4 and Table 4). Frequent MAPK pathway mutations have been previously reported in stage 4 NBs [14]. A high frequency of RAS-MAPK pathway mutations has also been recently reported in relapse NBs [45]. Together with these studies, our current results indicate that mutations in MAPK pathway components are associated with an aggressive phenotype and poor outcome for NB, suggesting that targeting the MAPK pathway might be a promising strategy against aggressive NBs. Additionally, we found that 9 genes with HiFI mutations were enriched in the Wnt signaling pathway (Figure 4 and Supplementary Table 3). Recently, roles for Wnt signaling in axon guidance, synapse formation and remodeling have been uncovered [46, 47]. Moreover, several lines of evidence show that SLIT/ROBO signaling coordinately regulates Wnt signaling activity [48, 49]. Taken together, our findings suggest that the axon guidance and Wnt signaling pathways might have a synergistic role in NB tumorigenesis and tumor progression, especially in *MYCN*-amplified NBs.

## MATERIALS AND METHODS

### Clinical samples

56 tumor/normal (blood leukocyte) paired NBs including 57 primary and 7 recurrent /metastatic tumor samples were subjected to WES analysis in this study. They comprised 5 stage 1, 5 stage 2, 6 stage 3, 35 stage 4 and 5 stage 4s cases. Of these, 51 tumors were sporadic and 6 had familial background coming from 4 pedigrees. All specimens were obtained from NB patients after informed consent at hospitals joining the Japan Neuroblastoma Study Group. Details concerning clinical information and genomic subgrouping are shown in Supplementary Table 1. All samples were subjected to histological review by a pathologist to confirm diagnosis and assess overall tumor content. *MYCN* amplification and DNA ploidy index were further confirmed by fluorescence *in situ* hybridization and flow cytometry, respectively. The Chiba Cancer Center Institutional Review Board approved the use of clinical samples in this study.

### Array CGH analysis and genomic subgrouping

Microarray-based comparative genomic hybridization (Array CGH) analysis was conducted for 343 NB samples using the Human Genome CGH Microarray Kit (44K or 244K, Agilent), following the manufacturer's protocol. In combination with their chromosomal aberration profiles including 1p loss, 11q loss and 17q gain as well as *MYCN* amplification, each tumor was categorized into genomic subgroups [20, 21]. Of those, 21 tumors in the Ss subgroup and 17 in the P1a subgroup, along with 18 tumors belonging to the other genomic subgroups including Sa (6), P1s (1), P2a (2), P3s (2), P4s (2), W1s (1) and W4s (4), were employed for WES analysis.

### Whole-exome sequencing and somatic mutation analysis

Quantified DNA samples from 64 tumors (57 primary tumors and 7 recurrent/metastatic tumors) and matched blood leucocytes were randomly fragmented to produce an average insert size of 200 bp. These fragments were subsequently amplified by ligation-mediated PCR (LM-PCR), purified, and hybridized to the Agilent SureSelect exome (50M) array for enrichment. Streptavidin bead-captured LM-PCR products were then evaluated using the Agilent 2100 Bioanalyzer to estimate the magnitude of enrichment. Each captured library was loaded onto one lane of the Illumina Hiseq2000 platform using 90-bp paired-end reads for high-throughput sequencing to ensure that each sample met the required depth of at least 75X.

Sequencing reads were aligned to a human reference genome (hg19) using Burrow-Wheeler Aligner [50]. Duplicate reads were marked by Picard [51]. We applied MutTect [52] to call somatic single-nucleotide variants. Mutations supported by less than four variant reads were filtered out. For detection of small indels, PIndel was used with default parameters [53]. Identified indels were labeled as somatic if no evidence for the event at the same loci was observed in matched blood DNA data. The remaining indels were subsequently manually inspected to eliminate those of low quality. All filtered mutations were annotated by ANNOVAR [54] and Oncotator [55] and then used for follow-up analysis.

Validation was performed using PCR/Sanger sequencing technology to verify the candidate mutations including those that were enriched in our pathway enrichment analysis, occurred in two or more cases, and were specifically identified in the Ss subgroup. Moreover, validated mutations were further tested in an independent NB sample set and 25 NB cell lines using Sanger sequencing.

### Evolution analysis

We used Recapseg and AlleiCapseg to call copy number changes of 64 NB tumors. The segment results are highly identical to those identified by array CGH analysis (Supplementary Figure 1). We then applied ABSOLUTE [56] to infer the absolute copy number for all tumors based on the relative copy number segments. The CCF was measured based on the local copy number of each segment and the allele frequency of each somatic SNV. Somatic mutations were divided into clonal mutations and subclonal mutations. A somatic SNV was classified as a subclonal mutation if it has ≥ 50% probability to be a subclonal mutation. Tumors with at least 40% of chromosomal arms exhibiting the absolute copy number ≥ 2 were classified as having undergone WGD.

Phylogenetic trees were constructed according to the criteria described by Brastianos, et al [57]. In brief, we inferred the trees based on the shared clonal mutations, private clonal mutations and private subclonal mutations. Shared clonal mutations were identified as mutations shared by primary and recurrent/metastatic tumors for each case. The length of each branch is proportional to the number of mutations on that branch.

### Statistical analysis

Statistical analysis was performed using R software (version R 3.0.1). Kaplan–Meier survival curves were drawn based on distinct genomic subgrouping. Survival distributions were compared using the log-rank test. Permutation testing was conducted to evaluate the association of the number of somatic mutations with various genomic aberrations in the context of *MYCN* amplification versus *MYCN* non-amplification or P1a versus Ss. Statistical significance was declared if the *P*-value was < 0.05.

## SUPPLEMENTARY MATERIALS FIGURE AND TABLES


