# Supplementary material for: Genomic analysis–integrated whole-exome sequencing of neuroblastomas identifies genetic mutations in axon guidance pathway
Source: Oncotarget. 2017 May 23;8(34):56684–97. doi: 10.18632/oncotarget.18079 (PMC5593593; doi:10.18632/oncotarget.18079)
Supplement: Supplementary file 1 [file oncotarget-08-56684-s001.pdf]

Genomic analysis–integrated whole-exome sequencing of neuroblastomas identifies genetic mutations in axon guidance pathway

SUPPLEMENTARY MATERIALS

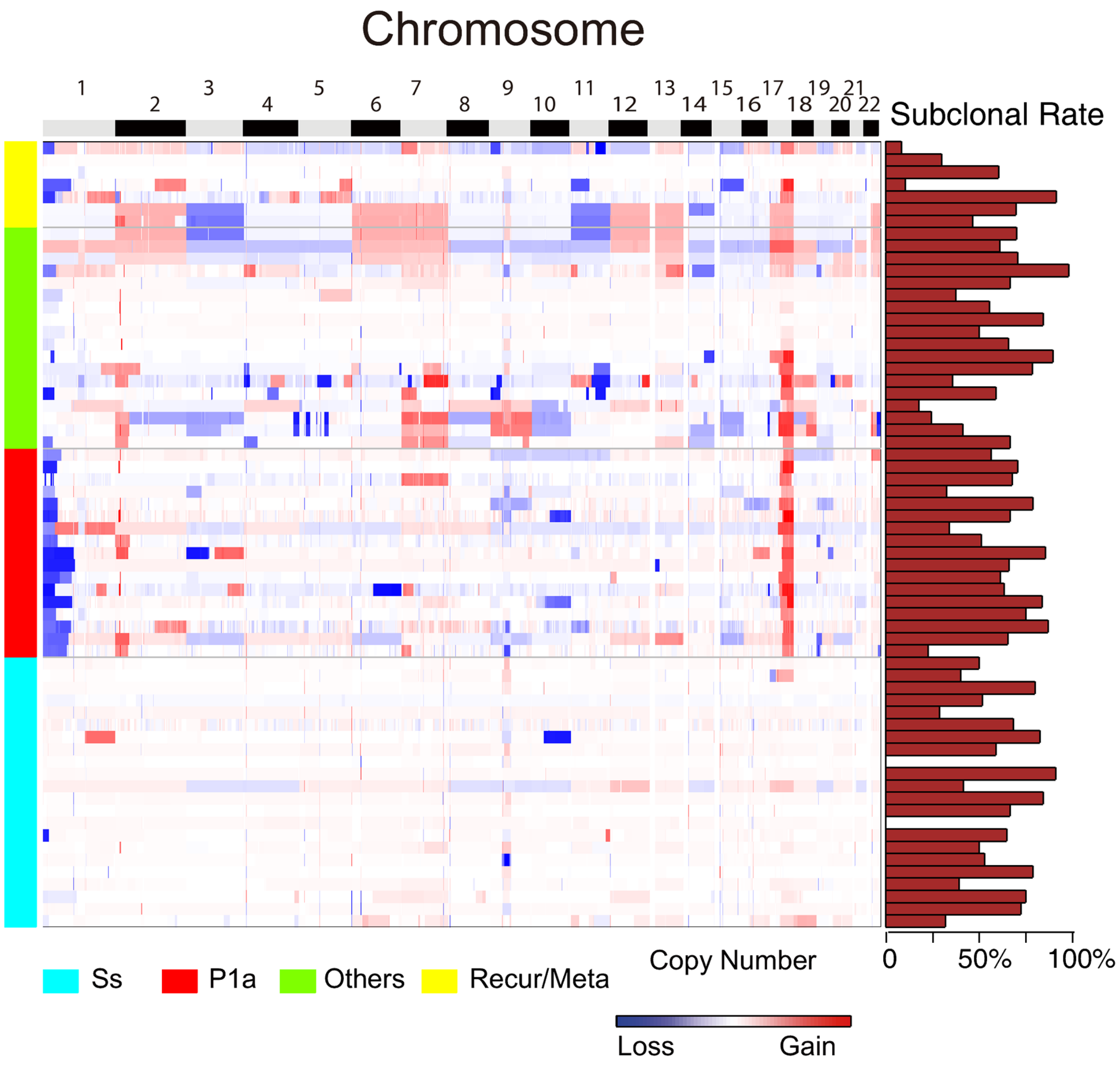

Supplementary Figure 1: Copy number alterations of 64 NB tumors. In the heatmap, SCNAs of each sample (vertical axis) are plotted along chromosomal locus (horizontal axis). The left bar shows the genome grouping for each tumor. The right bar graphs show the subclonal mutation rates.

Supplementary Table 1: Clinical information of 64 NBs.

See Supplementary File 1

Supplementary Table 2: List of somatic mutations of 64 NBs.

See Supplementary File 2

Supplementary Table 3: KEGG pathway analysis results.

See Supplementary File 3
